# Biocultural tourist experience in Romania’s High Nature Value rural landscape: Application of an extended Theory of Planned Behavior

**DOI:** 10.1371/journal.pone.0324444

**Published:** 2025-05-20

**Authors:** Ruxandra Malina Petrescu-Mag, Hamid Rastegari, Tibor Hartel, Kinga-Olga Reti, Dacinia Crina Petrescu

**Affiliations:** 1 Department of Environmental Science, Faculty of Environmental Science and Engineering, Babes-Bolyai University, Cluj-Napoca, Romania; 2 Doctoral School “International Relations and Security Studies”, Babes-Bolyai University, Cluj-Napoca, Romania; 3 Department of Rural Development Management, Faculty of Agriculture, Yasouj University, Yasouj, Iran; 4 Department of Hospitality Services, Faculty of Business, Babes-Bolyai University, Cluj-Napoca, Romania; University of Malta, MALTA

## Abstract

The present study looks at the interface of environment and culture, where High Nature Value (HNV) rural landscapes serve as a scene for a new type of tourism that creates a “biocultural tourist experience”. This study aimed to develop and test a new conceptual framework that explains the tourists’ behavioral intention formation by using an extended Theory of Planned Behavior. We used PLS-SEM on the data collected from a large-scale representative sample of 1007 Romanian people to predict their Intention to have a biocultural tourist experience in a HNV rural landscape. The study’s results verified that the model had a satisfactory predictive power and highlighted the crucial role played by Attitude, Subjective Norms, Perceived Behavioral Control, Trust, and Values in shaping the Intention to engage in a biocultural tourist experience in a HNV rural landscape. Notably, HNV Awareness does not influence Attitude or Intention. The research uncovers the intricate elements that constitute the biocultural tourist experience, where nature and culture intertwine, creating a narrative that is as much about the natural intrinsic value of the land as it is about the lived traditions of its inhabitants and the human-nature connection. The study suggests enhancing the attractiveness and accessibility of biocultural tourist experiences. Stakeholders promoting these experiences in HNV rural landscapes should prioritize authenticity, sustainability, and alignment with cultural and environmental values to boost credibility and encourage participation.

## 1. Introduction

The connection between individuals and nature is crucial for achieving personal well-being, particularly within the context of tourism. Research consistently shows that a strong connection to nature is linked with higher levels of hedonic (pleasure-based) and eudaimonic (meaning-based) well-being [1,2]. This relationship is characterized by increased positive affect, vitality, and life satisfaction [3]. Regular visits to natural areas and participation in simple nature-related activities are strong predictors of improved well-being [4].

The present research is framed within biocultural discourses where rurality has transitioned from prioritizing agricultural production to emphasizing rural areas as “sites of consumption” [5], particularly for urban visitors. The increased interest in this consumption function of rural areas leads to the rise of post-productivism values of rural areas, centered on health and leisure activities [6]. These activities coexist with agricultural ones within rural areas, bringing both opportunities and challenges.

The intricate relationship between agriculture and biodiversity underscores the importance of sustainable farming practices in preserving ecological balance and promoting resilient ecosystems [7], where human contribution is crucial. Sustainable agriculture safeguards biodiversity and serves as a cornerstone of tourism, where travelers engage with local communities and ecosystems [8]. Biocultural tourism can contribute to the preservation of natural and cultural values, including High Nature Value (HNV) farming practices. HNV farming is central to bridging agriculture and biodiversity, thus creating a harmonious environment that benefits agricultural activities, biodiversity conservation [9], and tourism. Andersen et al. [10] defined HNV farmland as those regions in Europe where agriculture serves as a primary, often predominant, land use. In those areas, agriculture contributes to or is connected to the highest biodiversity in terms of species and habitats and may also feature the existence of species recognized for European conservation efforts or a combination of both factors.

In recent years, there has been increasing recognition of the various public goods and ecosystem services that HNV farmland offers [11]. The multifaceted contributions of HNV to our environment and well-being range from food supply to carbon sequestration, flood mitigation, a wide range of biodiversity, and opportunities for tourism [11]. Some of the most important farming areas in Europe are in Romania, and they are known for their HNV. The HNV farmland covers about 5 million hectares, or about 30% of all land used for farming in the country [12]. Unfortunately, biodiversity-rich farming landscapes face several threats that have escalated in the past century [13]. One is the intensification of farming that significantly reduced farmland biodiversity [14]. Another is land abandonment [15]. Last but not least, under current socio-economic conditions, HNV farmlands tend to be unattractive to rural communities due to their low profitability and substantial demands for manual labor [13,16]. HNV farming practices emphasize the importance of maintaining diverse landscapes, fostering a harmonious relationship between agriculture, biodiversity, and the promotion of sustainable tourism [11].

**Fig 1:**
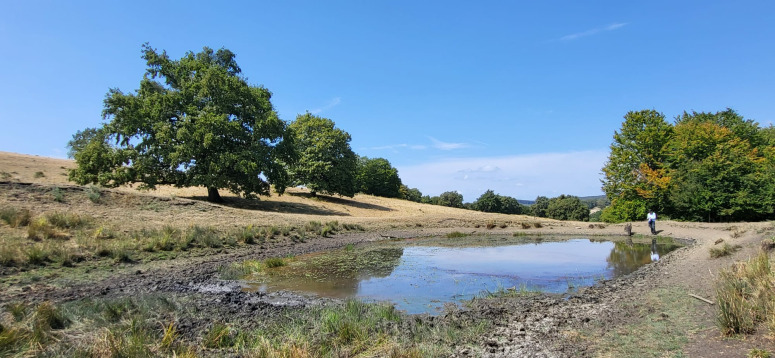
Ancient oak wood-pasture with high biocultural heritage and natural values, Saschiz Commune, Mures County (central Romania), 2024; Source: Authors’ personal archive.

**Fig 2:**
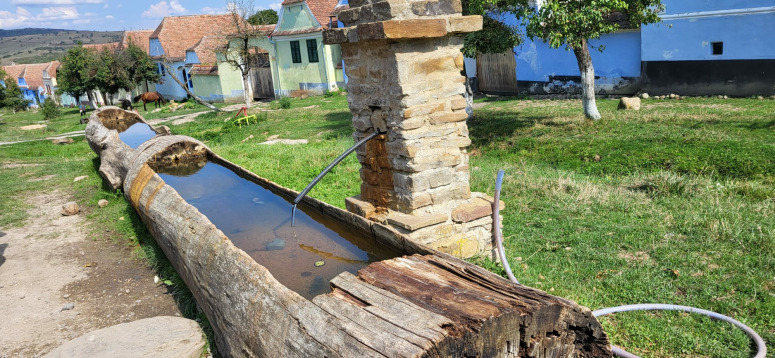
Traditional drinking trough for cattle, now valued as a local identity, heritage, and visitor attraction point in Viscri Village, Brasov County (central Romania), 2024; Source: Authors’ personal archive.

**Fig 3:**
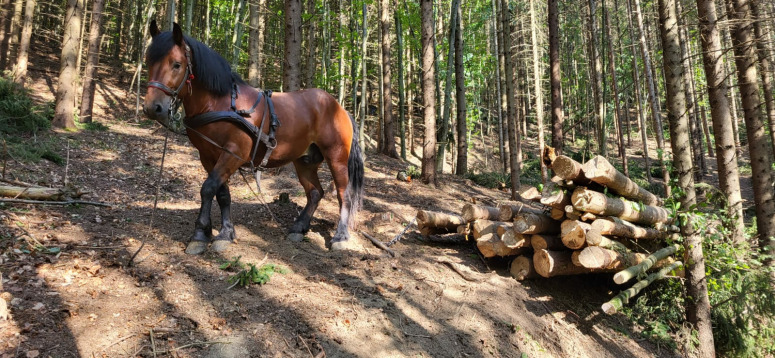
Traditional logging with the horse in the Eastern Carpathians, Covasna County, 2024; Source: Authors’ personal archive.

**Fig 4:**
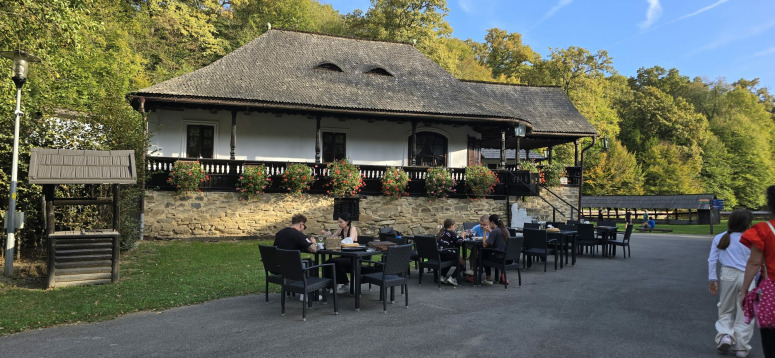
“Astra” National Museum Complex: old inn and a well, Sibiu County (central Romania), 2024; Source: Authors’ personal archive.

The HNV areas offer a mix of natural beauty and cultural heritage that aligns perfectly with the goals of studying tourist motivations to visit them. In addition to the prominence of HNV farming in Romania, the country’s touristic potential makes Romania a key case study for exploring tourist interest in these landscapes. This potential is highlighted by several country features. First, Romania has a diverse natural and cultural landscape, ideal for biocultural tourism. Romania is known for its pristine forests and wildlife in the Carpathian Mountains [17] and the biodiversity-rich Danube Delta. Rural areas in Romania combine traditional agricultural landscapes with high biodiversity [18]. The practice of traditional farming and living offers authentic cultural experiences that tourists are increasingly seeking [19,20]. Romania has a strong heritage of rural and cultural traditions and a wealth of UNESCO-listed cultural sites, such as the Painted Monasteries of Bucovina and Saxon villages in Transylvania [21]. These cultural treasures, often located in or near HNV areas, offer a unique combination of cultural and natural tourism experiences [21]. Second, Romania offers both connectivity via road networks and economic accessibility. Third, the increasing popularity of rural, ecological, and cultural types of tourism and their potential to contribute to the development of rural communities and nature preservation [22,23] make Romania a timely and relevant case study for examining tourist motivations related to HNV areas. Several examples of rural areas in Romania that can provide a biocultural tourist experience thanks to their beautiful landscapes, well-preserved nature, and cultural heritage are included in Figs 1–4.

Visitors can appreciate and contribute to the preservation of these interconnected ecosystems through actions, support, and advocacy, thus becoming conservation partners alongside local people, authorities, and conservation organizations. However, not all forms of tourist involvement benefit nature and local communities. It is crucial to understand the factors that influence tourists’ sustainable behaviors and adopt the appropriate measures to encourage them.

Previous studies used the Theory of Planned Behavior (TPB) to explore eco or cultural tourism intention and also used extended versions of the TPB by incorporating awareness, knowledge, values, awareness of consequences, and other variables [24] but they did not merge them into one model in a biocultural tourist experience context. The current study distinguishes from the previous studies in three ways that include: 1) the current study may be the first one to define the “biocultural tourist experience”; 2) this study merged TPB constructs (Attitude, Subjective Norm, Perceived Behavioral Control, and Intention) and Awareness, Knowledge, Values, and Trust in the Authenticity of tourist experience into one theoretical framework; and 3) the current study applied the extended TPB model in the “biocultural tourist experience” context. The definition of the biocultural tourist experience and further conceptual clarifications are included in section “2. Conceptual clarifications and theoretical background” (to allow the space necessary for a comprehensive presentation of this concept).

The present study looks at the interface of environment and culture, including social interaction tourists–community, where HNV rural landscapes serve as an example of a new type of tourism that we call “biocultural tourist experience” (defined in section 2). The discourse around biocultural relationships has evolved, emphasizing the intricate co-evolutionary connection between humans and their environments [25,26].

The present study seeks to reveal the nuanced factettes that constitute the biocultural tourist experience, where nature and culture intertwine, creating a narrative that is as much about the natural intrinsic value of the land as it is about the lived traditions of its inhabitants and the human-nature connection. The study aims to respond to the research question: “What factors influence Romanian people’s Intention to have a biocultural tourist experience in a HNV rural landscape?”. Consequently, the main objective of the study is to identify the factors that influence the Intention to have a biocultural tourist experience in the Romanian rural landscapes of HNV. To this end, we analyzed the effects of Awareness, Knowledge, Values, Trust in the Authenticity of tourist experience, Attitude (ATT), Subjective Norms (SN), and Perceived Behavioral Control (PBC) on the Intention to have a biocultural tourist experience in a HNV rural landscape.

In this context, the main contributions and novelty of this study lie in defining the biocultural tourist experience and developing and testing a conceptual framework that explains the tourists’ behavioral intention formation. Understanding the intricate interplay between the psychological constructs and the intention to take sustainable actions is fundamental to designing effective strategies that promote sustainable tourism. By exploring the attitudes and behaviors of people, we hope to provide valuable information that can inform policymakers, local communities, and tourism operators in their efforts to improve the sustainability of these biocultural tourist experiences. We also aim to contribute to the sustainable development of rural communities, where preserving the natural and cultural heritage inherent in these landscapes should come first. Therefore, we open the debate on the transformative potential of biocultural tourist experiences, not only as a catalyst for economic growth in rural areas but also as a mechanism to preserve HNV landscapes.

## 2. Conceptual clarifications and theoretical background

We define the term “biocultural tourist experience” as an immersive encounter that combines elements of local authenticity which includes natural components (such as air, water, plant and animal species, land, and landscapes), cultural heritage (such as historical vestiges and traditional practices and products), and social interaction. It involves engaging with a community to experience and appreciate the interconnectedness of biological diversity, cultural practices, and community interaction. Unlike other forms of tourism that may focus solely on either natural or cultural aspects, the biocultural tourist experience uniquely blends these elements to create a holistic encounter, where nature, culture, and human interaction are interwoven. Although the word “biocultural” was previously used in other contexts, such as anthropology (where it highlighted the influence of social environments on human health [27]), the “biocultural tourist experience” has different connotations.

Our definition of the biocultural tourist experience differs from the established concept of ecotourism, as encompassed by Fennell’s [28] six key attributes to achieve a “genuine” ecotourism experience. Although ecotourism emphasizes nature-based activities, preservation, education, sustainability, distribution of benefits, and responsibility, our definition of a biocultural tourist experience extends beyond the environmental domain. Our concept of a biocultural tourist experience intentionally broadens the scope beyond environmental factors. It expressly underlines the integration of cultural heritage and social interaction, portraying a more holistic and interconnected approach to sustainable tourism. In essence, our definition of “biocultural tourist experience” emphasizes the importance of preserving natural ecosystems in tandem with embracing the cultural heritage and lived traditions. The focus on social interaction underscores the active participation of tourists in local customs and community dynamics, highlighting how these interactions contribute to the authenticity and meaningfulness of the tourist experience. This focus on social interaction adds a distinction from ecocultural tourism, which is concerned with the general combination of ecological and cultural aspects [29]. By making these distinctions, we aim to create a niche within sustainable tourism that acknowledges and promotes the intrinsic link between nature, culture, and community in the pursuit of a meaningful and responsible tourist experience.

A comprehensive understanding of the biocultural experience in tourism and conservation is enriched by previous studies that investigated the interaction between nature, culture, and community engagement, thus contributing valuable information to the evolving discourse in this field. Breakey and Breakey [30] looked into tourism as a way to improve biocultural conservation experiences. They considered biocultural tourism to be a type of tourism that included learning opportunities and experiences with nature and/or cultural heritage. It is a type of tourism that prioritizes biocultural conservation by ethical considerations to encompass new ecological and/or cultural phenomena. Through participatory action research, Kaulen-Luks et al. [31] investigated the development of biocultural heritage within an Important Agricultural Heritage System in the southern Andes. In Serbia, both local communities and tourists valued biocultural experiences, considering the natural beauty and cultural heritage essential to the local economy and as attractions for tourists [32,33]. Kaulen-Luks et al. [31] also highlighted the concept of biocultural memory, which they defined in line with Nazarea’s [34] understanding, as knowledge, practices, and shared beliefs that arise from the interaction between local actors and their land. These studies collectively suggest that while biocultural tourism shares elements with ecotourism and cultural tourism, it represents a distinct form of engagement that deeply values both ecological and cultural dimensions, shaped through direct and meaningful social connections.

Our study employs the Theory of Planned Behavior (TPB), a well-established cognitive theory that considers that an individual’s decision to engage in a specific behavior can be predicted by their intention to engage in that behavior [35]. TPB suggests that intentions are determined by three variables: Attitude, SN, and PBC. Attitudes toward a behavior are the positive or negative evaluation of performing that behavior. SN show how people view the other people’s ideas about them engaging in a specific behavior, or the perceived social pressure (from the people important to them, e.g., family, friends, and colleagues) to engage or not to engage in a behavior. PBC is the extent to which people believe that they can control their behavior. This depends on their perception of internal factors, such as their ability and determination to perform a specific action, and external factors, such as the resources and support available to them. The theory argues that people’s PBC can affect their intentions to behave in a certain way, i.e., the more control they think they have over their behavior, the stronger their intention to perform it is. Behavioral intention considers the degree to which people are willing or plan to perform a certain behavior.

**Fig 5:**
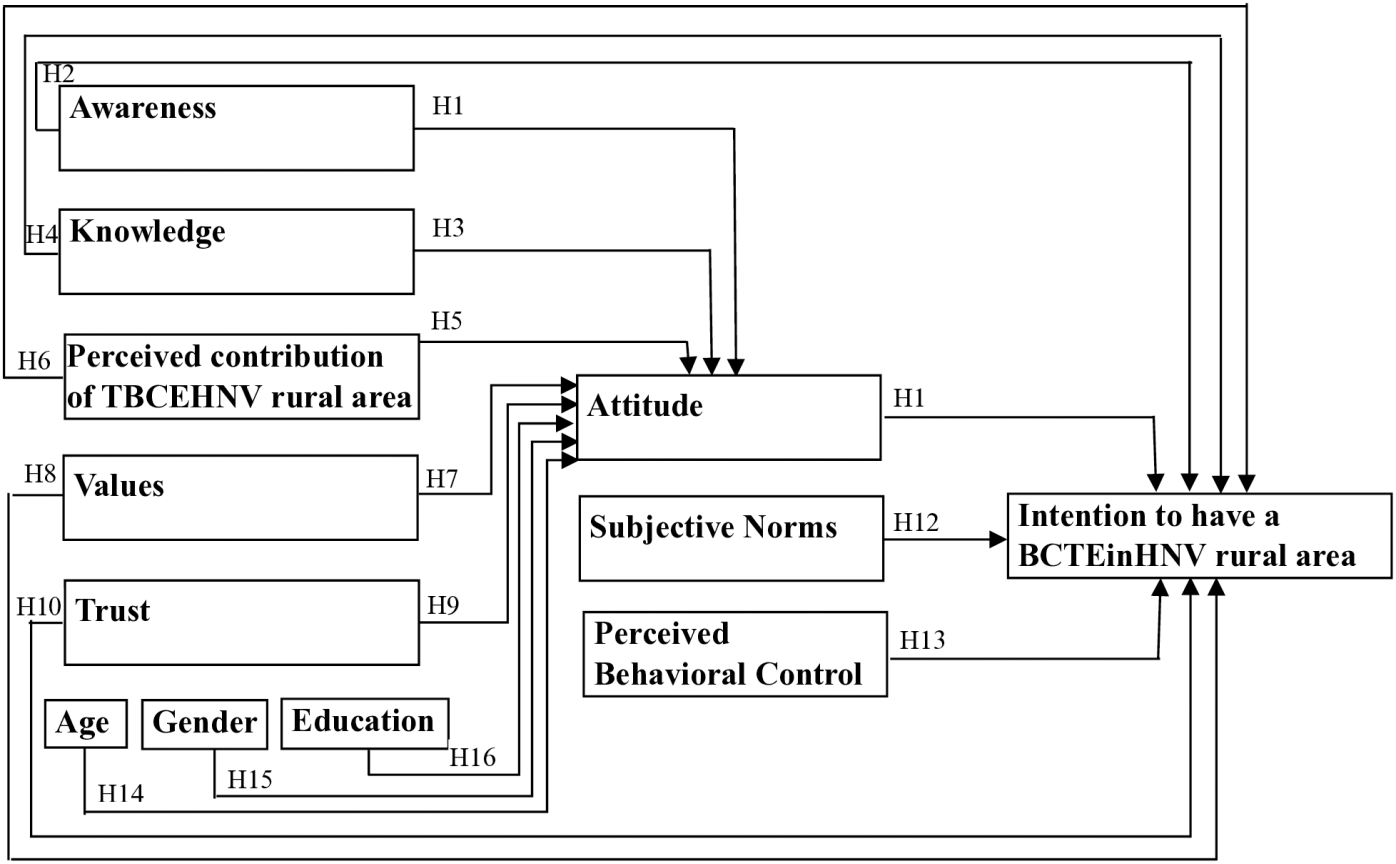
Hypothesized model. Legend for Fig 5: BCTEinHNV rural landscape = biocultural tourist experience in a HNV (High Nature Value) rural landscape; Awareness = awareness of the existence of HNV areas concept; Knowledge = factual knowledge about HNV areas characteristics; Contribution of BCTEinHNV = Positive effects of biocultural tourist experience in a HNV rural landscape to ecological, social, and economic dimensions of the HNV area; Values = respondents’ values regarding HNV areas; Trust = trust in the authenticity of a biocultural tourist experience in a HNV rural landscape; Attitude = attitude towards having a biocultural tourist experience in a HNV rural landscape.

The TPB has been widely used and extended in the tourism field to improve the understanding of the tourists’ intention to engage in tourism-related activities [24]. Despite the frequent application of the TPB in behavior research [36,37], including ecotourism, to the best of our knowledge, it was not related to biocultural tourism or biocultural tourist experience. Verma et al. [38] used TPB to predict the intention of young Indians to visit green hotels and found that Attitude, SN, and PBC significantly and positively influence the intention to visit green hotels. Zhang et al. [39] showed that Attitude, SN, and PBC influenced tourists’ and hikers’ pro-environmental behavior intention. Following Ajzen’s model, we hypothesized that Attitude, SN, and PBC positively influence the Intention to have a biocultural tourist experience in a HNV rural landscape (H11, H12, and H13, Fig 5; Table 1).

**Table 1:** Hypotheses of the study and the basic literature with the evidence that makes the hypothesis proposal reasonable*.

Hypothesis	Basic literature with the evidence that makes the hypothesis proposal reasonable
H1: Awareness has a positive effect on Attitude.	[43]
H2: Awareness has a positive effect on the Intention to have a biocultural tourist experience in a HNV rural landscape.	[44]
H3: Knowledge has a positive effect on Attitude.	[50]
H4: Knowledge has a positive effect on the Intention to have a biocultural tourist experience in a HNV rural landscape.	[47]
H5: Contribution of biocultural tourist experience in a HNV has a positive effect on Attitude.	[57]
H6: Contribution of biocultural tourist experience in a HNV has a positive effect on the Intention to have a BCTEinHNV rural landscape.	[57]
H7: Values have a positive effect on Attitude.	[59,65]
H8: Values have a positive effect on the Intention to have a biocultural tourist experience in a HNV rural landscape.	[59]
H9: Trust in the Authenticity of a biocultural tourist experience in a HNV rural landscape has a positive effect on Attitude,	[74]
H10: Trust in the Authenticity of a biocultural tourist experience in a HNV has a positive effect on the Intention to have a biocultural tourist experience in a HNV rural landscape.	[73]
H11: Attitude has a positive effect on the Intention to have a biocultural tourist experience in a HNV rural landscape.	[35]
H12: SN has a positive effect on the Intention to have a biocultural tourist experience in a HNV rural landscape.	[35]
H13: PBC has a positive effect on the Intention to have a biocultural tourist experience in a HNV rural landscape.	[35]
H14: Age has a positive effect on Attitude.	[78]
H15: Women have a more favorable Attitude.	[79,80]
H16: Education has a positive effect on Attitude.	[81]

*Please see the legend of Fig 5 for the full name of the variables.

While research has demonstrated the efficacy of the TPB in predicting intentions and behaviors, scholars noted some weaknesses and proposed incorporating additional variables to improve the predictive power of the original model [40]. Numerous studies applied modifications and revisions, using models with new variables, substitute variables, or a combination of TPB and other models [41]. For example, a study on an extended TPB concerning the anticipation of intentions to conserve biodiversity observed that awareness significantly influences the attitude toward behavioral intentions related to biodiversity conservation [42]. Awareness also influences the attitude of tourists toward environmentally responsible behavior [43] and fruit retail store managers’ intention to perform fruit waste management [44]. The cited studies underscore the importance of interventions to raise awareness in promoting pro-biodiversity behaviors. Awareness and knowledge of the environment could influence recycling behavior in a study by Ramayah et al. [45]. On this basis, we propose that H1: Awareness has a positive effect on Attitude toward having a biocultural tourist experience in a HNV; and H2: Awareness has a positive effect on Intention to have a biocultural tourist experience in a HNV rural landscape (Fig 5, Table 1).

Soliman [46] found that knowledge of travel motivation was a predictor that could stimulate the interest of tourists to revisit Egypt. Similarly, the inclination of Iranian farmers towards environmentally friendly behavior, such as biodiversity conservation and vermicomposting was influenced by knowledge, showing the relevance of knowledge in shaping their intentions and behavior [36,47]. Studies also noted that a positive shift in environmental attitudes appears as environmental knowledge increases ([48,49] cited by [50]). Liu et al. [50] demonstrated that knowledge positively affected environmental attitude. HNV knowledge includes factual knowledge about HNV structures, functions, and processes [51–53] (concept defined similarly to environmental knowledge as understood by Schahn and Holzer [54], and Boerschig and De Young [55], cited by [50]). Thus, we proposed that H3: Knowledge has a positive effect on Attitude; and H4: Knowledge has a positive effect on Intention to have a biocultural tourist experience in a HNV rural landscape (Fig 5 and Table 1).

Andereck et al. [56] argued that a stronger perception of tourism’s positive effects could lead to greater support for this activity. Çelik & Rasoolimanesh [57] observed that a positive perception of the tourism effect generates positive cost-effect attitudes and support for tourism development. In light of these findings, we considered that the Contribution of the biocultural tourist experience in a HNV rural landscape to nature, society, and the economy (the pillars of sustainable development and highlighted in various studies about ecosystem services, such as [58]) could have an impact on Attitude and Intentions and we formulated H5: Contribution of biocultural tourist experience in a HNV rural landscape has a positive effect on Attitude; and H6: Contribution of biocultural tourist experience in a HNV rural landscape has a positive effect on Intention to have a biocultural tourist experience in a HNV rural landscape.

Values contribute to the motivational aspect of the TPB. Values are attitudes towards abstract entities and have the power to influence attitudes and behaviors in various contexts [59]. Values can be powerful motivators, influencing an individual’s commitment to a particular behavior [60]. Several studies indicated that specific values (e.g., egoistic and social-altruistic) positively influence sustainable behaviors [61] such as climate change [62] or conservation behavior [63,64]. Gansser and Reich [65] found a strong influence of egoistic and altruistic values on the attitude toward sustainable behavior. Relational values are “preferences, principles, and virtues about human-nature relationships” [66]. The concept of relational values transcends the traditional dichotomy of instrumental and intrinsic values by emphasizing the significance of people’s relationships with nature and their connections with others mediated by nature [67]. Consequently, relational values play an essential role in constructing human–nature interactions [66–68]. These studies lead us to propose the following hypotheses about relational values (as they were mentioned in [69]): H7: Values have a positive effect on Attitude; and H8: Values have a positive effect on Intention to have a biocultural tourist experience in a HNV rural landscape.

Trust was found to be another important determinant of behavioral intention and behavior [70–72]. A positive and significant correlation between trusting the hotel and the intention to make a booking was reported in a study on German tourists [73]. When individuals trust a person, entity, or system, they are more inclined to engage in positive behavioral intentions and actions. Furthermore, Wu & Chen [74] placed trust as an important antecedent of attitude. Authenticity improves the quality of tourists’ experience [75], increases the chances that tourists visit a destination again [76], and enhances identities, well-being, and sustainability of host communities [77]. The above research argument leads us to propose these research hypotheses: H9: Trust in the Authenticity of the biocultural tourist experience in a HNV rural landscape has a positive effect on Attitude; and H10: Trust in the Authenticity of the biocultural tourist experience in a HNV rural landscape has a positive effect on Intention to have a biocultural tourist experience in a HNV rural landscape.

Finally, many studies included demographics in their TPB models. For example, Hong et at. [41] demonstrated the significant positive effect of demographics on the attitude in the hospitality sector (about participation in a restaurant health promotion program). In this context, we hypothesized that H14: Age has a positive effect on Attitude; H15: Women have a more favorable Attitude; and H16: Education has a positive effect on Attitude.

Based on the above, we propose the model presented in Fig 5, with the hypotheses detailed in Table 1. We capitalized the first letter of each variable (e.g., Attitude) when we referred to the variables of this study to differentiate them from the variables used in other studies. Awareness and the items of Knowledge were dichotomous variables, and the rest of the variables were measured on 7-point scales (except for demographics) (please see Table A.1., S1 Appendix, for details).

## 3. Methodology

### 3.1. Sample selection and description of the constructs

The study used a survey with 1007 valid questionnaires. A specialized company collected the responses. The general population included Romanian citizens over 18 years of age who were fit to travel. All the subjects provided appropriate written informed consent. This was obtained by the specialized company that collected the data. Details about the ethical considerations and GDPR are included at the end of Table A.1., S1 Appendix. The study received the Ethical approval no 92/ 06.01.2023 from Babes-Bolyai University. The questionnaire was administered online, data was collected between 20.12.2023 and 20.01.2024, and the sample was representative at the country level regarding gender, age, and geographical distribution (considering the nine development regions of Romania). The average age of the sample was 39.6 years, and 51.7% of the sample were women (Table A.1., S1 Appendix). The response rate was 19.4%. The questions addressed the six selected variables and demographics (Table A.1, S1 Appendix). The language of the questionnaire was Romanian because it was applied to a sample of Romanian people. A panel of three experts confirmed the questionnaire’s face validity. The questionnaire was pretested on 34 people to ensure the wording was appropriate and adjusted as needed. The pretest included feedback questions where we asked the respondents to tell if there were unclear questions, words, or meanings and if they suggested improvements. Within the questionnaire, the respondents received the definitions of the concepts of HNV rural landscape, biodiversity, and biocultural tourist experience to ensure a common understating of these concepts among respondents.

### 3.2. Measurement model

Structural equation modeling (SEM) is a group of statistical methods that estimate relationships among sets of observed variables that form a model [82]. PLS-SEM is designed as a “causal-predictive” approach, and it aims to explain the variance in the model’s dependent variables [83]. This study is about predicting target constructs and finding effect relationships. The PLS-SEM allows for the estimation of complex models with many constructs. Other recent studies about tourism have also used PLS-SEM [84]. For these reasons, the appropriate procedure for testing the hypotheses in this research model and predicting peoples’ Intention to have a biocultural tourist experience in a HNV rural landscape is structural equation modeling with partial least squares, PLS-SEM.

## 4. Results

### 4.1. The measurement model

Model fit measures in PLS are normed fit index (NFI) and standardized Root Mean Square Residual (SRMR). These values obtained from the PLS analysis were equal to 0.91 (>0.90) and 0.072 (<0.08), respectively, which indicate that the model highly fits the data [85–87]. In addition, the constructs were evaluated by outer loadings, composite reliability (CR), average variance extracted (AVE), Fornell-Lacker criterion, and heterotrait-monotrait (HTMT) criteria, and they are indicated in Tables 2–4. All outer loadings of reflective constructs are greater than 0.6, which shows that all outer loadings are suitable. Outer loadings reflect the convergence between the items in each construct. Furthermore, all CR and AVE values have significant levels, which means that all items in each construct have an acceptable relationship (Table 2). These values correspond to the values proposed by Hair et al. [86] and Garson [85].

**Table 2:** Measurement of the reliability of the constructs.

Constructs and items	Outer loading	Cronbach’s Alpha	CR	AVE
Attitude (ATT*)		0.909	0.925	0.580
ATT1	0.574			
ATT2	0.799			
ATT3	0.710			
ATT4	0.764			
ATT5	0.815			
ATT6	0.798			
ATT7	0.802			
ATT8	0.790			
ATT9	0.771			
**Age**	1	1	1	1
**Awareness of HNV (Awareness)**	1	1	1	1
**Perceived contribution of BCTEinHNV**		0.885	0.929	0.813
Contribution BCTEinHNV Econ	0.877			
Contribution BCTEinHNV Enviro	0.899			
Contribution BCTEinHNV Socio	0.928			
**Education**	1	1	1	1
**Gender**	1	1	1	1
**Knowledge**	1	1	1	1
**Intention to have a BCTEinHNV (Intention)**		0.956	0.968	0.884
INTENTION1	0.926			
INTENTION2	0.953			
INTENTION3	0.944			
INTENTION4	0.939			
**PBC**		0.846	0.898	0.689
PBC1	0.865			
PBC2	0.857			
PBC3	0.892			
PBC4	0.690			
**SN**		0.870	0.911	0.719
SN1	0.851			
SN2	0.871			
SN3	0.863			
SN4	0.804			
**Trust in the authenticity of a BCTEinHNV (Trust)**	1	1	1	1
**Values**		0.942	0.952	0.712
Values1	0.816			
Values2	0.809			
Values3	0.837			
Values4	0.857			
Values5	0.840			
Values6	0.889			
Values7	0.858			
Values8	0.843			

**Fig 6:**
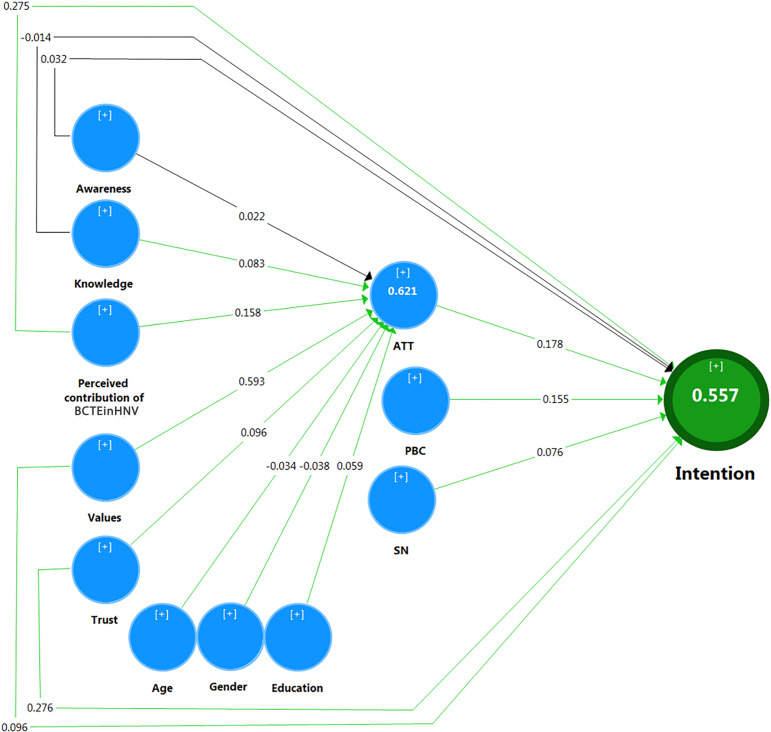
Measurement structural model of the Intention of having a biocultural tourist experience in a HNV rural landscape (black line = not significant effect; green line = significant effect; R squares are in circles).

*Please see the legend of Fig 5 for the full name of the variables. The abbreviations indicated between the paratheses are also used in Fig 6.

**Table 3:** Results of Fornell-Lacker Criterion.

Construct	1	2	3	4	5	6	7	8	9	10	11	12
(1) ATT	0.762											
(2) Age	0.063	1										
(3) Awareness	0.11	-0.074	1									
(4) Education	0.037	-0.041	-0.07	1								
(5) Gender	-0.074	0.207	-0.028	-0.038	1							
(6) Intention	0.612	-0.008	0.139	0.013	-0.036	0.94						
(7) Knowledge	0.265	0.266	-0.093	-0.005	0.024	0.153	1					
(8) PBC	0.706	0.029	0.134	-0.019	-0.026	0.598	0.143	0.83				
(9) Perceived contribution of BCTEinHNV	0.585	-0.005	0.119	-0.02	-0.053	0.635	0.209	0.549	0.902			
(10) SN	0.663	0.046	0.15	-0.027	-0.054	0.556	0.081	0.692	0.519	0.848		
(11) Trust	0.547	0.039	0.107	-0.076	0.013	0.628	0.229	0.529	0.648	0.508	1	
(12) Values	0.756	0.141	0.112	-0.02	-0.035	0.59	0.235	0.788	0.58	0.769	0.561	0.844

Note: The values in the diagonal show the square root of the average variance extracted (AVE). Off-diagonal values represent the unchanged values of the inter-factor correlations. Please see the legend of Fig 5 for the full name of the variables.

**Table 4:** Heterotrait-monotrait (HTMT) distribution of constructs.

Construct*	ATT	Age	Awareness	Education	Gender	Intention	Knowledge	PBC	Perceived contribution of BCTEinHNV	SN	Trust
ATT											
Age	0.066										
Awareness	0.114	0.074									
Education	0.040	0.041	0.070								
Gender	0.073	0.207	0.028	0.038							
Intention	0.635	0.018	0.142	0.018	0.037						
Knowledge	0.270	0.266	0.093	0.005	0.024	0.155					
PBC	0.770	0.057	0.149	0.035	0.063	0.663	0.179				
Perceived contribution of BCTEinHNV	0.632	0.055	0.127	0.023	0.056	0.690	0.221	0.630			
SN	0.713	0.048	0.161	0.029	0.058	0.606	0.089	0.803	0.588		
Trust	0.553	0.039	0.107	0.076	0.013	0.642	0.229	0.572	0.689	0.543	
Values	0.789	0.144	0.116	0.023	0.037	0.621	0.241	0.876	0.633	0.846	0.577

*Please see the legend of Fig 5 for the full name of the constructs (variables).

Table 3 shows the most consistent items of each latent variable with the same variable. This information indicates the validity and reliability of the analysis. In addition, heterotrait-monotrait (HTMT) criteria shown in Table 4 have values below the threshold of 0.8 suggested by Hair et al. [86]. This means that all items belong to their own construct and deeply differ from other constructs [86]. We used PLSpredict to assess a model’s predictive power. The minority (or the same number) of indicators in the PLS-SEM analysis yields greater prediction errors compared to the naïve LM benchmark, which indicates a medium predictive power.

### 4.2. The structural model

The total effects and “R^2^” values are indicated in Table 5 and Fig 6. Attitude has a total effect (*β* = 0.178) and a significant effect (*p* < 0.001; see Table 5) on the Intention to have a biocultural tourist experience in a HNV rural landscape. This positive effect indicates that an increase in Attitude is associated with an increase in Intention to have a biocultural tourist experience in a HNV rural landscape.

**Table 5:** Total effects of independent constructs on the Intention to have a biocultural tourist experience in a HNV rural landscape.

Path from→to	Total effect	Standard Deviation	T Statistics	P Values
ATT →Intention	0.178***	0.038	4.649	0.001
Age → ATT	-0.034*	0.019	1.759	0.079
Age →Intention	-0.006 ^**NS**^	0.004	1.501	0.134
Awareness →ATT	0.022 ^**NS**^	0.019	1.178	0.239
Awareness →Intention	0.032 ^**NS**^	0.020	1.589	0.113
Education →ATT	0.059***	0.020	2.999	0.003
Education → Intention	0.010**	0.005	2.285	0.023
Gender → ATT	-0.038*	0.019	1.941	0.053
Gender → Intention	-0.007*	0.004	1.727	0.085
Knowledge → ATT	0.083***	0.019	4.409	0.001
Knowledge → Intention	-0.014 ^**NS**^	0.023	0.614	0.539
PBC → Intention	0.155**	0.050	3.101	0.002
Perceived contribution of BCTEinHNV → ATT	0.158***	0.032	4.878	0.001
Perceived contribution of BCTEinHNV → Intention	0.275***	0.045	6.094	0.001
SN → Intention	0.076*	0.042	1.813	0.070
Trust → ATT	0.096***	0.029	3.345	0.001
Trust → Intention	0.276***	0.044	6.316	0.001
Values → ATT	0.593***	0.027	21.865	0.001
Values → Intention	0.096*	0.053	1.819	0.069

Note:

***p < 0.001;

**p < 0.01;

*p < 0.1; NS: not significant.

Age has a negative (*β* = –0.034) and significant (sig. = 0.079; see Table 5) effect on Attitude about having a biocultural tourist experience in a HNV rural landscape. This negative coefficient indicates that an increase in age is associated with a decrease in Attitude about having a biocultural tourist experience in a HNV rural landscape.

Gender has a negative and significant effect on Attitude (*β* = -0.038, sig. = 0.058). The negative effect indicates that men have a less favorable Attitude towards having a biocultural tourist experience in a HNV rural landscape compared to women.

Education positively affects Attitude, and the coefficients are β = 0.059, sig. = 0.003. This positive effect indicates that an increase in education is associated with more favorable Attitude towards having a biocultural tourist experience in a HNV rural landscape.

Similarly, the Knowledge about HNV area functions has a positive (*β* = 0.083) and significant (sig.=<0.001) effect on the Attitude toward having a biocultural tourist experience in a HNV rural landscape. This means that the more knowledgeable people are, the more favorable their Attitude is. However, Knowledge does not have a direct significant effect on the Intention of having a biocultural tourist experience in a HNV rural landscape.

PBC has a positive and significant effect on the Intention to have a biocultural tourist experience in a HNV rural landscape (*β* = 0.155, sig. = 0.002). This means that an increase in the PBC is associated with an increase in the Intention of having a biocultural tourist experience in a HNV rural landscape.

The perceived contribution of biocultural tourist experience in a HNV rural landscapes to the economic, ecological, and cultural state of the HNV area has a positive effect on Attitude (β = 0.158, sig. = 0.001) and Intention of having a biocultural tourist experience in a HNV rural landscape (β = 0.275, sig. = 0.001). In other words, the higher the perceived contribution of the biocultural tourist experience in a HNV rural landscape is, the more favorable and the stronger the Intention to visit is.

SN have a positive and significant effect on Intention of having a biocultural tourist experience in a HNV rural landscape (*β* = 0.076, sig. = 0.070). This means that an increase in the SN is associated with an increase in the Intention of having a biocultural tourist experience in a HNV rural landscape.

Trust in the authenticity of a biocultural tourist experience in a HNV rural landscape has a positive effect on Attitude (β = 0.096, sig. = 0.001) and Intention of having a biocultural tourist experience in a HNV rural landscape (β = 0.276, sig. = 0.001). These positive effects indicate that an increase in Trust in the authenticity of a biocultural tourist experience in a HNV rural landscape is associated with an increase in both Attitude and Intention of having a biocultural tourist experience in a HNV rural landscape.

The Values have a positive effect on Attitude (β = 0.593, sig. = 0.001) and Intention to have a biocultural tourist experience in a HNV rural landscape (β = 0.096, sig. = 0.069). These positive effects indicate that an increase in Values is associated with an increase in both Attitude and Intention to have a biocultural tourist experience in a HNV rural landscape.

According to Table 5, the Awareness construct does not have a significant effect on the Attitude and Intention to have a biocultural tourist experience in a HNV rural landscape.

Regarding the structural model, the PLS-SEM method was applied to analyze Stone–Geisser’s Q^2^, R^2^, and path coefficients to observe the predictive accuracy and power of the model, and the strength of the relationship between the constructs on the determined paths. Table 6 indicates that the value of Q^2^ in the form of cross-validated redundancy for the model’s endogenous constructs is positive, with a value of 0.460, revealing that the model has predictive accuracy [86]. The obtained R^2^ for Intention is 0.557, indicating the model has high predictive power [88] and that 55.7% of the variance of the Intention to have a biocultural tourist experience in a HNV rural landscape is explained by the model. Similar R^2^ values were obtained in other studies in tourism and environmental fields that used the TPB (e.g., 0.475 in [89]; 0.48 in [40]). The R^2^ for Attitude shows that 61.8% of the variance of the Attitude is explained by the Perceived contribution of biocultural tourist experience in a HNV rural landscape, Knowledge, Age, Education, Gender, Trust, and Values (Fig 6).

**Table 6:** Q^2^, R^2^, and R^2^_Adjusted_ of the model.

Construct	SSO	SSE	Q^2^ (=1-SSE/SSO)	R^2^	R^2^_Adjusted_
Intention	4,028.00	2,173.330	0.460	0.557	0.554
Attitude	9,063.00	6,161.919	0.320	0.621	0.618

## 5. Discussion

The study sheds light on the interplay of Awareness, Knowledge, Values, Trust, Attitude, SN, and PBC, which shape the peoples’ Intention to have a biocultural tourist experience in a HNV rural landscape. With most (13 of 16) of the proposed hypotheses confirmed (Table 7) and around 60% of the variance in the Intention explained by the selected variables, stakeholders interested in promoting biocultural tourist experience in a HNV rural landscapes can rely on the model to understand the determinants of the people’s Intention.

**Table 7:** Overall view of the verification status of the hypotheses.

Hypothesis	Verification status of the hypotheses
H1: Awareness has a positive effect on Attitude.	Not confirmed
H2: Awareness has a positive effect on the Intention to have a biocultural tourist experience in a HNV rural landscape.	Not confirmed
H3: Knowledge has a positive effect on Attitude.	Confirmed
H4: Knowledge has a positive effect on the Intention to have a biocultural tourist experience in a HNV rural landscape.	Not confirmed
H5: Contribution of biocultural tourist experience in a HNV rural landscape has a positive effect on Attitude.	Confirmed
H6: Contribution of biocultural tourist experience in a HNV rural landscape has a positive effect on the Intention to have a biocultural tourist experience in a HNV rural landscape.	Confirmed
H7: Values have a positive effect on Attitude.	Confirmed
H8: Values have a positive effect on the Intention to have a biocultural tourist experience in a HNV rural landscape.	Confirmed
H9: Trust in the Authenticity has a positive effect on Attitude,	Confirmed
H10: Trust in the Authenticity has a positive effect on the Intention to have a biocultural tourist experience in a HNV rural landscape.	Confirmed
H11: Attitude has a positive effect on the Intention to have a biocultural tourist experience in a HNV rural landscape.	Confirmed
H12: SN has a positive effect on the Intention to have a biocultural tourist experience in a HNV rural landscape.	Confirmed
H13: PBC has a positive effect on the Intention to have a biocultural tourist experience in a HNV rural landscape.	Confirmed
H14: Age has a positive effect on Attitude.	Partially confirmed: the effect is significant, but the direction is negative, being opposed to the hypothesized one
H15: Women have a more favorable Attitude.	Confirmed
H16: Education has a positive effect on Attitude.	Confirmed

The findings highlight a crucial role played by Attitude in shaping the Intention to engage in a biocultural tourist experience in a HNV rural landscape. The significant and positive relationship between Attitude and Intention indicates that fostering a favorable Attitude toward the biocultural tourist experience in a HNV rural landscape can drive individuals’ desire to participate in these experiences, underscoring the importance of shaping perceptions through targeted interventions. In Australia, the attitude toward embarking on a vacation centered around wine experiences significantly impacts one’s Intention toward such an experience [90]. To understand the travel intentions of tourists during the COVID-19 pandemic, Wang et al. [91] employed TPB and found that attitude partially mediates travel intentions, with age moderating the relationship between SN and travel intention. In our case, age negatively and significantly affects Attitude. The negative impact of age on Attitude toward having a biocultural tourist experience in a HNV rural landscape highlights a generational divide in perceptions. Younger individuals tend to exhibit more favorable Attitude toward these experiences compared to their older counterparts, highlighting the need for age-specific strategies. Nonetheless, it is worth noting that overall – across both younger and older age groups – respondents expressed favorable Attitudes, with average item scores related to Attitude ranging between 5.6 and 6.0 (Table A.1, S1 Appendix). This result aligns with previous research, reinforcing the idea that age plays a significant role in shaping attitudes toward tourism experiences, particularly in ecotourism [78]. Similar to the current study, in Italy, younger people were more motivated to choose sustainable accommodation if the location was easily accessible by public transportation and had few tourists [92]. As a result, tourism management strategies should be adapted to reflect the unique environmental concerns and interests of various age groups, ensuring inclusivity and long-term sustainability. One possible explanation for the current study results is that younger generations in Romania are increasingly exposed to global conversations about environmental protection and cultural heritage through channels such as social media, international travel, and formal education. This exposure has likely cultivated a stronger appreciation for nature and cultural heritage, which, in turn, may have led to the desire to engage with and preserve such environments, and a more favorable attitude toward visiting places that offer natural and cultural experiences. Romania’s rich natural landscapes and diverse cultural heritage present a valuable opportunity to engage the younger segment, whose preferences align closely with experiences centered around authenticity, sustainability, and cultural exploration. Moreover, younger people may perceive nature- and culture-based tourism as more affordable and accessible alternatives to luxury or international travel, further enhancing their favorable attitudes toward such experiences.

Surprisingly, Awareness does not yield a significant effect on Attitude and Intention. This indicates that mere familiarity with the concept is not enough to shape individuals’ Attitude and Intentions toward having a biocultural tourist experience in a HNV rural landscape. On the one hand, in line with these findings, Antimova et al. [93] discussed the existence of the awareness – attitude/ behavior gap related to pro-environmental travel behavior and argued that the lack of influence on attitude and intention could be explained by examining the gap at different levels: individual, interpersonal, and community levels. They concluded that individual-level theories offer a better explanation for the gap than the other two theories and the most common reasons for the gap were skepticism, distrust, fatalism, and pursuing self-interest and comfort. On the other hand, contrary to the present findings, other studies highlighted the influential role of awareness [43,44]. One possible interpretation of the current results is that while awareness plays a role in certain contexts, it may be less effective in influencing more complex or specialized decisions, such as engaging with HNV rural landscapes. This suggests that in the context of Romania’s rural landscapes, a more targeted approach may be necessary to engage individuals, rather than relying solely on awareness. Unlike previous research where awareness prompted revisits, the distinctiveness and complexity of HNV areas require strategic emphasis on the variables with significant effect, such as Knowledge, Values, Trust, PBC, rather than simply providing information about HNV areas in efforts to stimulate the Intention to visit. From a practical perspective, this can be seen as an encouraging finding, as participants demonstrated a positive Attitude towards having a biocultural tourist experience in a HNV rural landscape and Intention, regardless of prior awareness of HNV [the majority of them did not hear about HNV previous to the survey (62%), but the total sample has positive Attitude (5.8 average score) and Intention (5.6 average score); Table A1, S1 Appendix]. Also, this result can indicate that Awareness alone may be insufficient to influence people’s Attitudes or Intentions. People need deeper knowledge or understanding of the significance of HNV areas and an emotional connection to develop a positive attitude toward visiting them. The significant and positive effect of Knowledge on Attitude (see Fig 6) further supports this assumption.

Knowledge emerges as a positive and significant determinant of Attitude, showcasing the importance of information in shaping Attitude towards having a biocultural tourist experience in a HNV rural landscape. Exploring the innovative motivations of travelers aiming to contribute to environmental conservation and examining the factors that influence their eco-conscious choices, Gautam [94] found that greater environmental knowledge leads to a more positive attitude towards environmentally friendly products, with international tourists showing higher awareness compared to domestic tourists. Despite the positive influence of Knowledge on Attitude, its direct impact on the Intention to engage in biocultural tourist experience in a HNV rural landscape is not significant. Other studies also concluded that while knowledge alone did not consistently predict behavior, possessing correct knowledge was important for overcoming psychological barriers such as unconsciousness and fear ([95,96] cited by [50]). Furthermore, it is unlikely to make wise environmental choices if one has incorrect or no knowledge ([97,98] cited by [50]). Our research indicates that possessing accurate Knowledge about HNV areas and the biocultural tourist experience in a HNV rural landscape can build engagement and make people feel personally connected or motivated to explore them and, as such, generate a positive Attitude. A similar lack of a significant effect of knowledge on behavioral intention was found in other studies [99]. The lack of a significant direct relationship may be explained by the fact that, in an era of abundant information, individuals might experience overload, leading to decision paralysis, thereby weakening the direct influence of knowledge on intention. However, there is an indirect effect. Thus, although Knowledge does not directly affect Intention, it contributes to the formation of a positive Attitude, which in turn influences Intention. This suggests that, in the end, individuals who have the right knowledge are more likely to Intend to visit a HNV rural landscape. Furthermore, the economic, social, and environmental contributions that tourists can make to HNV areas should be actively promoted to educate people about their potential to effect change, contribute meaningfully, and benefit in return.

The perceived Contribution of biocultural tourist experience in a HNV rural landscape exhibits positive effects on both Attitude and Intention, suggesting that individuals are more inclined to engage when they perceive an effect.

Values are generally stable constructs and can be challenging to change, requiring substantial effort, time, and research to understand how they can be influenced [100]. A more feasible approach to influencing Intention in the short term may be to strengthen existing Values that support it. This can be achieved by enhancing confidence in these Values, showing support from opinion leaders, and highlighting the relevant benefits they provide. In line with this, a study by Verma et al. [38] explored how different values (biospheric, egoistic, altruistic) and a sense of responsibility influenced consumers’ intentions to visit green hotels in India. It was found that biospheric values were particularly influential in shaping attitudes, and individuals with a higher level of ascribed responsibility tended to develop a positive attitude toward green hotels, which subsequently affected their intention to visit. Similarly, Fauzi et al. [101] showed that environmental values (e.g., concern and commitment) significantly impact the attitude toward visiting a green hotel in Malaysia. Hou and Wu [102] further demonstrated the significance of deeply ingrained environmental values, both conscious and subconscious, in shaping attitudes and intentions toward visiting green hotels. In Romania, the increase in the appreciation of rural, ecological, and cultural types of tourism [23] is proof that cultural heritage and natural beauty are appreciated by tourists. Individuals who prioritize cultural, community, and environmental values are likely to develop a positive attitude toward visiting culturally and naturally significant destinations, such as HNV rural areas. The high average scores obtained by the Values variable in this study (5.8 average score; Table A.1., S1 Appendix) and their positive effect on attitude and intention are an indication that they may lead individuals to feel a stronger personal motivation or obligation to experience and support such places. Ecotourism and heritage tourism are sectors where values play a significant role [103–105]. Investigated people, who are driven by strong Values, will seek meaningful experiences that align with their ethical or personal beliefs and are not just a leisure activity. Value-driven tourism can lead to transformational experiences, where visitors feel they are contributing to something greater, such as environmental conservation or cultural preservation. Values can thus, strengthen Romanians’ positive attitude and intention to visit.

Trust in the authenticity of these experiences also emerges as a significant influencer, indicating that establishing credibility is a key consideration in promoting such experiences. Providing proof of the authenticity of the biocultural tourist experience in a HNV rural landscape (e.g., presenting the history of the place, with traditions, natural elements, etc.) can enhance people’s Trust, which positively influences Attitude and Intention. Research on social capital revealed that generalized trust significantly incentivizes individuals to participate in environmentally beneficial activities [106]. As shown by Marcias [106], establishing trust, mainly through generalized trust and knowledge of environmental issues, is a significant catalyst for encouraging participation in environmentally beneficial activities. In China, authenticity perceived by ecotourists had a positive effect on their revisiting intentions [107]. Furthermore, perceived authenticity influenced values and these supported tourists’ intention to revisit and their engagement in sustainable behavior [107]. As such, tourists’ Authenticity perception and their Values related to environmental preservation or cultural respect are likely to not only visit these places but also engage in sustainable practices while there (e.g., minimizing waste, respecting local customs, and supporting local economies).

PBC emerges as a significant driver of Intention, underscoring the importance of the perceived ability of individuals to control their actions and influence their decision to have a biocultural tourist experience in a HNV rural landscape. Previous studies have indicated a significand and positive correlation between PBC and the intention of tourists to travel (e.g., [108,109]). Similarly, in a study dedicated to coastal tourism, positive emotional well-being and PBC positively influence the intention of tourists to revisit a coastal tourism destinations [110]. Information on how to find details about an HNV area, how to organize the trip, or how to get to the place can improve people’s Trust in their ability to have a biocultural tourist experience in a HNV rural landscape, i.e., their PBC.

The positive and significant impact of SN on Intention, revealing the amplitude of social influences in shaping peoples’ decisions. This underscores the potential efficacy of community-based initiatives and collaborative efforts to promote biocultural tourism, where people are likely to be influenced by the actions and attitudes of their social circles.

The Intention to visit a biocultural site may depend on the socio-cultural background of the tourists. In less rural countries, there may be less cultural connection to rural life, and thus, rural tourism might be seen as more novel or exotic. In countries with a strong urban culture, rural tourism could be marketed differently, emphasizing escapism or eco-tourism over cultural roots. In more rural countries, the motivation may be linked to national tourists’ ties to their national identity and pride. Therefore, it is important to signal the particular features of the Romanian context because the results should be interpreted considering the local context.

Romania has a high percentage of rural land (96% of Romania’s surface, in 2015; [111]) and population (43.9%; [112]). Also, 70% of the total number of urban settlements are small towns that maintain some features of the rural context (with less than 20000 inhabitants, most of them having between 5000 and 10000 inhabitants; they comprise about 10% of the country’s population) [113]. This large rural population often maintains strong ties to local traditions, crafts, and agricultural practices, which could drive an intrinsic interest in biocultural tourism, either as tourists or providers. The familiarity with rural life among Romanians might lead to a higher intention to visit rural destinations, as these areas may evoke nostalgia, cultural pride, or personal connections to heritage. Additionally, biocultural tourism might be seen as an accessible and culturally rich experience.

The study can be relevant for readers from more as well as less urbanized countries because it can inform interventions that respond to similar challenges. A literature review on rural tourism in developed and developing countries found that both contexts faced similar challenges, with the internal challenges being the most worrying, and social and political ones rating the highest in both contexts [114].

Several theoretical and practical implications can be highlighted. First, the proposed research model offered a deeper understanding of tourists’ Intention to have a biocultural tourist experience in a HNV rural landscapes. Although previous studies applied the TPB or an extended version to predict intentions, their studies used a different set of variables (only some of the variables included here and/ or others; e. g., attitude and motivations in [89]; awareness of consequences and connection to nature in [42]). This study theoretically provided a more comprehensive perspective on the tourists’ intention by incorporating more variables in the original TPB model, enhancing our understanding of how personal factors interact to influence tourists’ intention, and offering good prediction power. Second, the direct and indirect role of the additional variable within the model provided high theoretical value to this study. Thus, it is worth highlighting the relevance for the model of Values, Trust in the authenticity of the biocultural tourist experience, and Knowledge. To the authors’ best knowledge, this study was the first to observe both the direct and indirect effect of Values and Trust on tourists’ Intention. Hence, sustainability and tourism researchers should consider Values, Trust, and Knowledge when they study tourists’ Intentions. The fact that, contrary to what was hypothesized and different from other studies [45] HNV Awareness did not have a significant influence on Attitude and Intention, shows that, regardless of their previous Awareness of this concept, people can be stimulated to visit HNV areas for the biocultural experience it provides. This finding is relevant because it offers us insight into the mechanism of intention formation.

From a practical perspective, the extended TPB model could provide a broader framework for tourism agencies, local administration, and the local community that offers tourist services to understand the tourists’ behavioral Intention. They should prioritize creating marketing strategies and communication campaigns that emphasize the authenticity of the biocultural tourist experience, highlighting the unique cultural and ecological aspects of HNV landscapes. Underlining the genuine connection between visitors and the local traditions, ecosystems, and values could enhance the perceived value of the experience and attract tourists who are motivated by sustainability and cultural immersion. Local communities should be empowered to share their stories and traditions to strengthen tourists’ trust in the authenticity of the experience. Specific actionable strategies could include developing community-led workshops or guided tours that involve locals sharing their knowledge of the land, traditions, and sustainable practices. This direct involvement can help build trust and showcase the authenticity of the experience. Also, providing clear and accessible information on how tourists can minimize their environmental impact and contribute positively to local conservation efforts.

Tourism agencies, local administration, and the local community should be aware of the importance of Values and Trust in the authenticity and focus on them as drives of Attitude and Intention formation. They should also acknowledge that Attitude, social influences from others (SN), and how people perceive their capacity to have the biocultural tourist experience in HNV (PBC) are factors that significantly contribute to tourists’ Intention. In practice, enhancing Trust in the authenticity of the experience could involve transparent communication about the conservation efforts in the HNV areas and the involvement of local communities in preserving their cultural and natural heritage. Efforts should be directed towards enhancing Trust in the authenticity of the experience and supporting the formation of human-nature connection values with the aim of shaping a positive Attitude towards the visit of a HNV area. Perception of social support from important peers and trust in their capacity to do the visit should also be supported. For example, before the travel, promotion of facilities and information of how to make the trip can enhance PBC. Information on the necessity and positive effect of tourists’ involvement, as well as the appreciation received can be provided during and post travel to improve SN and influence the formation of Values.

## 6. Conclusion

### 6.1. Main findings

The study advocates that the human contribution to maintaining HNV farming delicate ecosystems is crucial for environmental conservation and the flourishing of nature-based tourism initiatives. The main findings can be grouped around several aspects, as follows: i) Human contribution to nature preservation: Human involvement is essential for preserving HNV farming ecosystems and supporting nature-based tourism; ii) Factors influencing Intention to have a biocultural tourist experience in a HNV rural landscape: Understanding the interplay between Attitude, SN, PBC, Intention, and other variables extending the TPB can help tailor interventions to boost interest in biocultural tourism. For example, we found that Attitude, SN, PBC, Perceived contribution of biocultural tourist experience in a HNV rural landscape, Values, and Trust in the authenticity of a biocultural tourist experience in a HNV rural landscape have a positive and significant effect on the Intention to have a biocultural tourist experience in a HNV areas; iii) Importance of Knowledge: Increasing Knowledge about the benefits, cultural significance, and environmental impact of biocultural tourist experiences is vital for shaping positive Attitudes (for example, by providing detailed information about these); iv) Enhancing PBC: Initiatives should focus on empowering individuals to make informed decisions and participate actively by offering diverse options, logistical support, and addressing access barriers; v) Leveraging SN: Utilizing social networks and community dynamics can enhance engagement by encouraging positive peer interactions and showcasing social support; vi) Awareness of the HNV areas does not influence Attitude and Intention; vii) Perceived contribution of biocultural tourist experience in a HNV rural landscape influence both the Attitude and Intention; viii) Building Trust in the authenticity of biocultural tourist experience in a HNV rural landscapes: Ensuring the authenticity of biocultural tourist experiences and aligning with cultural and environmental Values is important to gain credibility and attract participants.

### 6.2. Limitations of the research

The results must be seen in the context of study limitations and future research directions are recommended. Although this study significantly improves our understanding of biocultural tourism and establishes a foundation for further research and practical applications in sustainable tourism development, it is important to recognize its limitations. One limitation concerns the use of Intention as a proxy for the actual behavior, considering that there are studies that indicated a gap between these two variables [115]. Despite this limitation, a substantial body of research, especially within the TPB, demonstrated that intention is a strong predictor of behavior ([116] cited by [117]). Consequently, intention was usually used as a practical and feasible indicator in tourism studies, particularly when actual behavior data is difficult or resource-intensive to collect. Additionally, the current study can be seen as a critical first step in understanding intentions. Future studies can build on it, incorporate behavioral measures, and examine the transition from intention to action. The study relied on data from Romanian respondents and results should be interpreted in this context. Additional testing in varied contexts would provide further evidence for the generalizability and robustness of the measurement model.

### 6.3. Perspectives for future investigations

Future research could build upon the present study’s findings by exploring additional psychological and contextual factors, such as emotions, place attachment, and environmental ethics, to deepen our understanding of the drivers behind biocultural tourist behavior. Longitudinal studies could assess how intentions evolve into sustained behaviors over time while examining the impact of social media, or community initiatives (e.g., village days, fairs) on shaping tourists’ attitudes. Expanding the conceptual model to include mediators, moderators, and perceptions of economic outcomes could provide nuanced insights into sustainable tourism motivations. Within the country, comparative analyses across different tourist groups and regions would help refine the framework’s applicability and reveal variations in how cultural and ecological dimensions influence behavior. Internationally, the model’s cross-cultural applicability remains a topic for future research. Future studies should replicate the model with more populations from different countries and cultural backgrounds, allowing us to compare and contrast the results and assess the broader applicability of the findings. For example, it will be interesting to observe in a comparative context if differences between countries with different degrees of rurality influence the Attitude and the Intention to have biocultural tourist experience in a HNV rural landscape. Additionally, a comparison between countries with similar rural areas can show if cultural and environmental differences influence Attitude and Intention. The analysis could also include other variables, such as the tourist profile of people and their sustainability awareness because it was observed that they were correlated. Thus, in Ireland, it was found that sustainability awareness decreased from individual trip tourists to coach trip tourists to cruise ship tourists, indicating the need for segment-specific sustainability governance [118]. Other relevant variables may be the geographic and economic contexts. The accessibility of HNV areas and their natural beauty may enhance the intention to visit, compared to countries where rural areas might be less accessible or less picturesque. Also, the motivations behind the Intention to visit can also differ: in countries like Romania or Greece (with larger rural areas), tourists may be seeking culture and natural beauty, while in countries with larger, more urbanized landscapes (e.g., the US), tourists might want to travel long distances and get away from city life, rather than a nearby cultural exploration. Furthermore, exploring local communities’ perspectives on biocultural tourism and its impact on their traditions and environment, based on a qualitative approach, could lead to more inclusive biocultural tourism strategies.

## Supporting information

S1 Appendix Table A1. (DOCX)
