# Supplementary material for: Biocultural tourist experience in Romania’s High Nature Value rural landscape: Application of an extended Theory of Planned Behavior
Source: PLoS One. 2025 May 20;20(5):e0324444. doi: 10.1371/journal.pone.0324444 (PMC12091816; doi:10.1371/journal.pone.0324444)
Supplement: S1 Appendix — (DOCX) [file pone.0324444.s001.docx]

**Appendix**

**Table A.1.** The variables investigated in the study, associated questions in the questionnaire, supporting references, and answers

| **Investigated variables** (IV= independent variable; DV=dependent variable) | **The question in the questionnaire** | **Answer options** | **Referenced literature** | **Answers (percentage of respondents / average value)** |
| --- | --- | --- | --- | --- |
| **1. Awareness** (IV) | a) Have you heard of areas with High Nature value (HNV) before this questionnaire? | 1= No, 2=Yes |  | 1= No; 62%  2= Yes; 38% |
| **2. Knowledge** (IV) | Which of the following statements is true? You can tick several options. Select the ones that seem true to you. [multiple choice]  a) Rural landscapes with HNV are located in the countryside where traditional agriculture is the main economic activity and a key factor in nature conservation.  b) Rural landscapes with HNV ensure the livelihood of many communities in Romania.  c) Rural landscapes with HNV offer a rich heritage in terms of agricultural and culinary traditions and customs.  d) Rural landscapes with HNV ensure clean air, water, soil and the protection of biodiversity.  e) The protection of rural landscapes with HNV does not depend on the continuation of traditional agricultural practices. (Reversed)  f) Intangible values ​​(aesthetic, cultural, historical) are as important as tangible values ​​(wood, agricultural products, etc.) in rural landscapes with HNV. | 1 = The answer was selected; 0= The answer was not selected | [51–53];  Authors’ contribution (for point f) | a) 60.4%  b) 48.7%  c) 67.8%  d) 59.0%  e) 24.9%  f) 42.3% |
| **3. Contribution of the biocultural tourist experience in a HNV rural landscape** (IV) | a) Do you think that the biocultural tourist experience in an HNV rural area contributes to protecting the local natural environment?  b) Do you think that the biocultural tourist experience in an HNV rural area contributes to the preservation of the socio-cultural values ​​of the area? (such as traditions, monuments, etc.)  c) Do you think that the biocultural tourist experience in an HNV rural area contributes to the economic development of the area? | 1= It contributes to a very small extent, ..., 7= It contributes to a very large extent | [58] | a) 5.5  b) 5.5  c) 5.8 |
| **4. Values** (IV) | Show your agreement to the following statements:  (i) Relational values ​​(at the collective level):  a) Rural landscapes of HNV are important for the local community living there.  b) Rural landscapes of HNV are important to Romanian citizens.  c) My presence in rural landscapes with HNV helps me connect with people (social cohesion).  d) To take care of the ecosystems in the rural landscapes with HNV represents an essential part of the care I have for the people who live in the rural landscapes with HNV (people of the present and the future) (social responsibility)  e) Taking care of all forms of life is a moral necessity for me (moral responsibility towards non-humans)  (ii) Relational values ​​(individual level)  f) Rural landscapes with HNV are important to me, and to who I am as a person (individual identity).  g) My concern for rural landscapes of HNV fulfills me and helps me lead a good life (eudaimonic stewardship)  h) Maintaining a balanced natural environment of rural landscapes with HNV is the right thing to do (principle/ value of the administration) | 1= Total disagreement, …, 7= Total agreement | [69] | a) 6.0  b) 5.9  c) 5.6  d) 5.6  e) 5.9  f) 5.8  g) 5.7  h) 6 |
| **5. Trust** **in the authenticity of a biocultural tourist experience in a HNV rural landscape offered by the local community** (IV) | a) How much confidence do you have in the authenticity of the biocultural tourist experience offered by the local community in a rural landscape with HNV? In other words, do you trust that the food is from local products, cooked according to traditional recipes, traditions are genuine and local, the natural landscape is with species of the area, etc.? | 1= I have no confidence at all, ..., 7= I have total confidence | Authors’ contribution | a) 5.6 |
| **6. Attitude** **(**IV) | a) How interested are you in visiting an HNV area in the next 12 months for the biocultural experience it offers you? | 1= Not at all, …., 7= Very much | [35] | a) 5.9 |
|  | How is a biocultural experience in HNV areas for you? | b) 1= It is very unpleasant for me, 2, 3, 4, 5, 6, 7= It is very pleasant for me  c) 1= It is very harmful for me, …., 7= It is very beneficial for me  d) 1= It is completely useless for me, …., 7= It is very useful for me  e) 1= It is completely uninteresting to me, …., 7= It is very interesting to me  f) 1= It is very stressing for me, …., 7= It is very relaxing for me  g) 1= It upsets me very much, …., 7= It makes me very happy |  | b) 6.0  c) 5.7  d) 5.6  e) 5.7  f) 5.7  g) 5.8 |
|  | Show your agreement to the following statements :  h) The biocultural tourist experience in rural landscapes with HNV allows you to learn about nature.  i) The biocultural tourist experience in rural landscapes with HNV allows you to learn about traditions, the history of the place, and the community in the area. | 1= Total disagreement, …, 7= Total agreement |  | h) 5.9  i) 5.9 |
| **7. Subjective Norms** (IV) | Show your agreement to the following statements:  a) People who are important to me think that it is beneficial for me to have a biocultural experience in HNV areas.  b) People who are important to me visit HNV areas for the biocultural tourist experience.  c) When I see that many people visit rural landscapes with HNV for the biocultural tourist experience, I will visit them also.  d) When the public authorities support visiting rural landscapes with HNV for the biocultural tourist experience, I will visit them, too. | 1= Total disagreement, …, 7= Total agreement | [35]  [36,37] | a) 5.7  b) 5.6  c) 5.5  d) 5.3 |
| **8. Perceived Behavioral Control** (IV) | Show your agreement to the following statements:  a) I believe that I can have a biocultural tourist experience in HNV rural areas.  b) Having a biocultural tourist experience in HNV rural areas depends on me.  c) For me, it is easy to have biocultural tourist experience in rural HNV areas.  d) I have everything it takes (money, time, transportation means, information) to have a biocultural tourist experience in HNV rural areas. | 1= Total disagreement, …, 7= Total agreement | [35] | a) 5.9  b) 5.8  c) 5.7  d) 5.0 |
| **9. Intention to have a biocultural tourist experience in HNV rural landscape** (DV) | a) Show how willing you are to visit in the next 12 months a rural landscape with HNV that offers a biocultural tourist experience. | a) 1= I am not at all willing to do this, ..., 7= I am very willing to do this | [35] | a) 5.7 |
|  | b) I intend to visit a rural landscape with HNV that offers a biocultural tourist experience. | b) 1= Totally disagree, …, 7= Totally agree |  | b) 5.7 |
|  | c) I plan to visit in the next 12 months a rural landscape with HNV that offers a biocultural tourist experience. | c) 1= I will definitely not plan this, …, 7= I will definitely plan this |  | c) 5.6 |
|  | d) I will make all the preparations in the next 12 months to visit a rural landscape with HNV that offers a biocultural tourist experience. | d) 1= I will definitely not do that, …, 7= I will definitely do that |  | d) 5.5 |
| **Demographic variables** |  |  |  |  |
| 10. Age (IV) | Open question |  |  | 39.6 years |
| 11. Gender (IV) | Please select your option | a) F b) M c) Other/ Prefer not to say |  | a) 51.7%  b) 48.3%  c) 0% |
| 12. Education (IV) | Please select your option | a) Up to 4 years of school  b) Between 5-8 years of school  c) Between 9-12 (or 13) years of school  d) Faculty  e) Master's degree  f) Doctorate |  | a) 0.4%  b) 1.4%  c) 32.8%  d) 45.3%  e) 17.9%  f) 2.3% |
| Details about GDRP and ethical considerations regarding how the respondents were informed about the study were included in the following text that was displayed (in Romanian) at the beginning of the questionnaire.  What is this survey about?  This survey is about tourism, High Nature Value rural landscapes, your views on biodiversity and other nature-related concepts.  How long does it take to complete this survey?  It takes about 35 minutes to complete.  What are we looking for?  We want to observe the factors influencing people's intention to have a biocultural experience in a rural landscape with High Natural Value. This study is part of the BIOTraCes project supported by the European Union's Horizon Europe. This study was approved by an independent ethics committee, namely the ethics committee of Babes-Bolyai University.  Rights and obligations of respondents  The beneficiary of your answers does not have access to your identification data. For them, your answers are anonymized. All information about your rights and obligations and GDRP data protection are included in your contract with XYZ [name of the data collection company; blinded to avoid promotion]. | | | | |
